# Development and Validation of an Oral Anticoagulation Knowledge Tool (AKT)

**DOI:** 10.1371/journal.pone.0158071

**Published:** 2016-06-28

**Authors:** Kehinde O. Obamiro, Leanne Chalmers, Luke R. E. Bereznicki

**Affiliations:** Pharmacy, School of Medicine, University of Tasmania, Hobart, Australia; Magna Graecia University, ITALY

## Abstract

**Background:**

Assessing and improving patients’ anticoagulation knowledge can lead to better treatment outcomes. While validated knowledge instruments exist for use in people taking warfarin, these tools are not necessarily applicable to patients taking direct-acting oral anticoagulants.

**Objective:**

To develop and validate an oral anticoagulation knowledge instrument that is applicable to all oral anticoagulant medications.

**Methods:**

Ten anticoagulation experts participated in the development of the Anticoagulation Knowledge Tool to ensure content validity. The knowledge instrument was administered to three groups of participants comprising of 44 pharmacists, 50 patients and 50 members of the general public. A subgroup of participants in the patient and pharmacist group were retested approximately 2–3 months after the initial testing. Statistical tests were conducted to determine the validity and reliability of the scale, and item analysis was used to determine the performance of individual questions.

**Results:**

The 28-item instrument developed had a scale content validity index of 0.92, supporting content validity. The pharmacist group’s mean score was significantly higher than that of the patient group, and the patient group scored significantly higher than the general public group (94% vs 62% vs 20%, respectively; p<0.001), supporting construct validity. Internal consistency reliability was acceptable with a Cronbach’s α value of > 0.7 across the three groups, and the test–retest reliability was confirmed with a Pearson’s correlation coefficient of 0.72 and 0.78 for the pharmacist and patient groups, respectively.

**Conclusion:**

The Anticoagulation Knowledge Tool is a valid and reliable instrument that can be used in routine clinical practice to assess patients’ anticoagulation knowledge.

## Introduction

Anticoagulants are widely used in the treatment and prevention of many thromboembolic disorders [1]. Patients’ knowledge of their medication and medical condition can affect treatment outcomes [2], and this becomes more critical in patients prescribed oral anticoagulants due to the narrow therapeutic indices of this class of medication, and the potentially devastating sequelae of both therapeutic failure and over-anticoagulation [3].

In the literature, attempts have been made to assess patient anticoagulation knowledge, and this has led to the development and use of a number of instruments in different settings. The earliest documented attempt to develop an instrument to evaluate patients’ anticoagulation knowledge was by Taylor et al, in which a scale was developed based on information available in a district hospital guideline for managing patients taking warfarin [4]. More recent attempts by researchers have developed scales based on the use of patient educational material, review of the literature and expert opinion using either open ended or multiple choice questions [5–7]. These scales have been used in a number of studies to establish the relationship between anticoagulation knowledge and treatment outcomes, and have yielded mixed results. Two of these studies have reported an association between adequate anticoagulation knowledge and positive treatment outcomes, [6, 7] while the other two have reported no association [5, 8]. A major limitation of these studies, however, is that none of them have employed the use of an instrument which has been psychometrically validated.

To date, only the anticoagulant knowledge assessment (AKA) by Briggs et al [9] and the oral anticoagulant knowledge test (OAK) by Zeolla et al [10] have been developed and validated with regard to both content and construct validity. However, both OAK and AKA have been designed to assess knowledge regarding vitamin K antagonists (VKAs) and are not applicable to the direct acting oral anticoagulants (DOACs). With the recent introduction of the DOACs (dabigatran, apixaban, rivaroxaban and edoxaban) into clinical practice, there is need for a validated instrument to assess patients’ knowledge of their anticoagulation therapy that applies to both the VKAs and the DOACs. The objective of this study was to develop and validate a knowledge instrument that can be used in assessing anticoagulation knowledge related to all the available oral anticoagulant medications.

## Methods

### Anticoagulation Knowledge Tool Development

We began by conducting a comprehensive review of the literature on patient anticoagulation knowledge, with additional information obtained from freely available patient educational material. The knowledge domain covered in the review of the literature included basic drug information, adverse drug effect, drug-drug interactions, drug monitoring and dietary issues. Similar information was then grouped to form a list of 56 items consisting of both open ended and multiple choice questions. The usefulness of each question in assessing anticoagulation knowledge was then discussed by the authors, after which the items were ranked on a scale of 1 to 5 (1 = strongly disagreed, 5 = strongly agreed) in terms of their relevance to anticoagulation knowledge. These rankings were used to eliminate irrelevant questions and create a 28-item draft instrument.

The items in the draft instrument were then discussed with 15 selected people from a non-medical background to ensure clarity of the sentences, simplify wording and to identify ambiguous and misleading terms. Items in the draft instrument were reworded based on the feedback received.

### Content Validity

Content validity refers to the degree to which a scale has an appropriate sample of items to represent the construct of interest [11]. To ensure content validity, the draft instrument was presented to 10 anticoagulation experts (8 pharmacists and 2 physicians) selected based on their work experience or research related to the use of oral anticoagulants. These experts were asked to rate the relevance of each item on the draft instrument on a four-point ordinal scale (1 = not relevant, 2 = somewhat relevant, 3 = quite relevant, 4 = highly relevant), and to suggest other items for the scale which may have been omitted. The content validity index for each item (I-CVI) and overall content validity of the scale (S-CVI) was then calculated using the method of Polit et al [11, 12]. In calculating the I-CVI, the rating scale was dichotomized, with ratings of ‘1’ and ‘2’ combined as not relevant, and ratings of ‘3’and ‘4’ combined as being relevant, while the S-CVI was calculated by determining the average of all the I-CVI values. Further, I-CVI values were translated into values of a modified kappa index (k*) so as to adjust for chance agreement among the experts participating in the content validity exercise. The modified kappa index was determined using the formula–k* = (I-CVI- pc)/ (1- pc), where pc refers to the probability of chance agreement among the experts and was computed using the formula for a binomial random variable, with one specific outcome (pc = [N! /A! (N—A)!] x 0.5^N; where ‘N’ = number of experts and ‘A’ = number of experts agreeing on relevance of an item). The average S-CVI of the scale was 0.92 with I-CVIs ranging from 0.6–1 and k* ranging from 0.5–1 (Table 1). The final instrument was divided into two sections–section ‘A’ and ‘B’, with section ‘A’ comprising general anticoagulation knowledge questions applicable to both the DOACs and VKAs, and section ‘B’ comprised of VKA-specific questions.

**Table 1:** Item and Scale Content Validity Indexes.

No	General questions	I-CVI	Modified kappa (k*) = (I-CVI-pc)/(1- pc)
1	What is the name of your anticoagulant medicine?	1.00	1.00
2	Why has your doctor prescribed you this medicine?	1.00	1.00
3	How does this medicine work in your body?	0.70	0.66
4	How many times a day do you need to take this medicine?	1.00	1.00
5	For how long do you need to take this medicine (for example, 3 months, and 6 months, life-long)?	1.00	1.00
6	Why is it important to take this medicine exactly as your doctor has told you?	1.00	1.00
7	Is it acceptable to take this medicine at different times as long as you take it on the required days?	1.00	1.00
8	Is it acceptable to double the next dose of this medicine if you miss a dose?	1.00	1.00
9	Is it possible that skipping one dose of this medicine could worsen your condition?	0.90	0.90
10	Is it appropriate to stop taking this medicine once you feel better?	0.90	0.90
11	Is it safe to take anti-inflammatory medicines like ibuprofen (Nurofen^®^ or Advil^®^) while you are taking this medicine?	1.00	1.00
12	Is it safe to take vitamin supplements and herbal medicines with this medicine without consulting your doctor?	0.90	0.90
13	Is there any benefit in taking more of this medicine than your doctor has told you to take?	0.80	0.79
14	Will drinking too much alcohol increase the risk of side effects with this medicine?	0.90	0.90
15	Is it necessary to inform a surgeon, dentist or other health professional that you are taking this medicine before undergoing surgery or a procedure?	1.00	1.00
16	Is it important that all the health care practitioners you see know that you are taking this medicine?	0.90	0.90
17	What is the most important side effect of this medicine?	0.80	0.79
18	Three signs of side effects that you should watch out for while taking this medicine are:	0.80	0.79
19	Three things you can do to reduce your risk of side effects are:	0.60	0.50
20	What is the best step to take if you accidentally take too much of this medicine?	1.00	1.00
	**Question specific to people taking warfarin**		
1	What is your target INR range?	0.90	0.90
2	What was your last INR reading?	1.00	1.00
3	Are routine INR tests necessary to know how well this medicine is working?	1.00	1.00
4	Is an INR value above your target range good for your general wellbeing?	1.00	1.00
5	Is it possible for INR values below your target range to be bad for your health?	0.90	0.90
6a	Is it possible for your diet to affect your warfarin therapy?	1.00	1.00
6b	If you answered ‘Yes’ above, list Three foods that can affect your anticoagulant therapy.	0.90	0.90
7	List one vitamin that can significantly affect your anticoagulant therapy.	0.80	0.79

pc (probability of a chance occurrence) was computed using the formula for a binomial random variable, with one specific outcome:

pc = [N!/A!(N—A)!]*0.5^N where N = number of experts and A = Number agreeing on good relevance.

k* = kappa designating agreement on relevance, k* = (I-CVI- pc)/(1- pc). k* of 0.4–0.59 (fair); 0.60–0.74 (good); and > 0.74 (Excellent). Average Scale-CVI = 0.92

### Pilot Study

In order to further ensure readability and comprehension, a pilot study was conducted in 13 participants (5 pharmacists, 3 patients and 5 members of the general public) representing the three groups to be compared. The results from the thirteen pilot studies participants were not included in the main study. Instructions on completing and returning the questionnaire were further revised based on the feedback obtained in the pilot study. The final instrument used in the study is available in S1 Appendix.

### Validation Study

Adults (aged > 18 years) who were able to read and complete the questionnaire independently were recruited into the validation study. All the participants in the validation study were recruited from Tasmania, Australia. Subjects were recruited into three groups comprising of a pharmacist (expert) group, patient group and general public group. The pharmacist group was expected to serve as the positive control while the general public group was expected to serve as the negative control. Pharmacists were recruited from a total of 26 community and hospital pharmacies; patients currently prescribed oral anticoagulants were recruited from 14 community pharmacies; and participants from the general public group were recruited from 12 public places (e.g. parks, bus stops and shopping malls). Participants from the general public group were eligible to participate in the study if they were not health professionals, patients prescribed oral anticoagulants and did not have close relationships with patients taking oral anticoagulants. A study information sheet for the study was provided to participants in the three groups which stated that anticoagulants are also called blood thinners, specifically to assist participants in the general public group who may be less familiar with the term ‘anticoagulant.’ Also, written informed consent was obtained prior to participation. Participants in the pharmacist and general public group were required to assume that they were currently taking an oral anticoagulant and answer the questions in both sections of the survey, while participants in the patient group were asked to respond to the survey based on the oral anticoagulant they had been prescribed by their physician. Patients who were prescribed any of the DOACS were required to answer the questions in section ‘A’ only, while patients who had been prescribed VKAs were asked to answer the questions in both sections. Participants in the pharmacist group were given the option of completing the test online or by using a paper format, while the other two groups completed the test by using only the paper format. Participants who preferred to use the paper format had the option of completing the survey on the spot, or return it using a reply paid envelope. The study protocol was reviewed and approved by the Tasmanian Health and Medical Human Research Ethics Committee.

### Validity and Reliability

Construct validity refers to the extent to which a measure adequately assesses the construct it purports to assess [13]. Construct validity was assessed using the contrasted group approach which involves identifying two or more groups of individuals who are expected to have different scores on the characteristics being measured by an instrument [13]. Using this approach, we hypothesised that the instrument would be sensitive to multiple levels of anticoagulation knowledge. Also, we expected the mean score of the pharmacist (expert) group to be higher than the mean patient group score, and the mean score of the patient group to be higher than that of the general public group.

Two reliability tests were conducted: test-retest reliability and internal consistency reliability. In order to ensure the instrument’s stability, a re-test was conducted at approximately 2–3 month after the initial test administration, a time period considered sufficient to reduce the impact of recall. All the participants in the pharmacist and patient group were eligible for re-test, with 32 participants in the patient group and 22 in the pharmacist group participating in the second test. Internal consistency reliability was also conducted across the three groups to ensure the inter-relatedness of the items in the instrument.

### Scoring

Scoring was done use a dichotomous scale, with a score of ‘1’ or ‘0’ for each correct answer or wrong answer, respectively. A maximum score of ‘1’ was allocated to each correct answer for all of the questions with the exception of item ‘6’, ‘18’ and ‘19’ in section ‘A’ and item ‘6b’ in section ‘B’. A maximum score of ‘2’ was obtainable for item ‘6’ in section ‘A’- (‘Why is it important to take this medicine exactly as your doctor has told you?’) - 1 mark each was allotted for answers related to the prevention of thromboembolism and answers related to minimising the risk of bleeding. For items ‘18’ and ‘19’- (‘three signs of side effects you should watch out for’ and ‘three things you can do to reduce your risk of side effect’, respectively) - 1 mark each was allotted for each correct sign of side effects to look out for and each correct approach to reduce the risk of bleeding. Lastly, for item ‘6b’ in section ‘B’ (‘list three foods that can affect your anticoagulant therapy’) - 1 mark each was allotted for three correct food substances mentioned. A maximum total score of ‘25’ was obtainable for patients taking the DOACs required to answer only section ‘A’ of the questionnaire, while a maximum total score of ‘35’ was obtainable for patients taking the VKAs (warfarin) required to answer both sections of the questionnaire. Final scores were presented as a percentage of correct answers for all the participants in the study.

### Statistical Analysis

Analysis of variance (ANOVA) was used in comparing the mean scores between the pharmacist, patient and general public groups, with p<0.05 considered statistically significant. Pearson's correlation was used in determining the correlation between the test and re-test scores for the pharmacist and patient groups, and a values between 0 and 0.49 were considered as ‘very low’ to ‘low’ correlation, while values between 0.5 and 1.0 were considered as ‘moderate’ to ‘very strong’ correlation. Cronbach’s alpha score was used in determining internal consistency reliability and across the three groups, with a score of 0.7 or greater considered acceptable [14]. Lastly, the relative difficulty of each item and the instrument’s ability to discriminate between groups was also analysed by determining the differences in the percentages of items correctly answered across the three groups. Statistical analysis was conducted using SPSS Version 22.0.

## Results

One hundred and forty-four participants, comprising 44 pharmacists, 50 patients and 50 members of the general public, participated in the validation study. Four surveys from the general public group were excluded from the analysis due to participants being either health professionals or having experience with the use of oral anticoagulants; one survey from the patient group was excluded from the final analysis because the patient was not taking an oral anticoagulant at the time of the study. Overall, the results of 139 participants were included in the analysis (Table 2).

**Table 2:** Demographic Characteristics.

	General public (n = 46)	Patients (n = 49)	Pharmacists (n = 44)
Male n (%)	28 (64)	34 (69)	14 (34)
Age in years (mean +/- SD)	38 ± 11	74 ± 12	34 ± 10
Highest education completed n (%)			
High school	14 (30.4)	18 (36.7)	NA
College	8 (17.4)	5 (10.2)	NA
Technical/ Vocational	5 (10.9)	9 (18.4)	NA
Bachelor degree	5 (10.9)	11 (22.4)	35 (79.5)
Post graduate	13 (28.3)	5 (10.2)	9 (20.5)
No formal education	1 (2.2)	1 (2.0)	0 (0)
Duration of oral anticoagulant therapy	NA	< 3 months 3 (6.1); 3–12 months 4 (8.2); 1–2 years 8 (16.3); > 2 years 32 (65.3); Not reported 2 (4.1)	NA

NA = Not applicable

The mean score for the pharmacist (expert) group was significantly higher than that of the patient group, and the patient group’s mean score was significantly higher than the general public group’s (p<0.001; Table 3). No statistically significant difference in score was observed between patients taking the VKAs and the DOACS (p>0.05). For internal consistency reliability, a value of 0.92 was obtained in the general public group, 0.71 in the patient group for the general anticoagulation questions and 0.87 for participants taking warfarin required to answer both sections ‘A’ and ‘B’, and 0.73 in the pharmacist group (Table 4). Test–retest reliability was confirmed with a Pearson’s correlation of 0.79 and 0.72 in the patient and pharmacist groups, respectively (Table 4). For the item analysis, item difficulty ranged from 0–100% across the three groups. The questions with the largest differences are listed in Table 5. Analysis of the patient group showed that patients taking the DOACs were less likely to view skipping a dose of prescribed oral anticoagulant as a problem compared to patients taking warfarin (p< 0.05). Neither the type of oral anticoagulant (warfarin or DOAC), nor the duration of anticoagulation therapy were associated with a significant difference in test score.

**Table 3:** Anticoagulation Knowledge Instrument Scores.

	General public (n = 44)	Patient (n = 49)	Pharmacist (n = 44)
Mean (%)	19.9 ± 16.4	62.0 ± 13.9	93.7 ± 6.9
Minimum (%)	2.9	31.4	65.7
Maximum (%)	62.9	91.4	100

Statistics F (2, 136) = 359.8; p < 0.0001

**Table 4:** Validity and Reliability Coefficients.

	General public	Patient	Pharmacist
Internal consistency (Cronbach alpha)	(n = 46) 0.92	(Section A, n = 49) 0.71; (Section A and B, n = 15) 0.87	(n = 44) 0.73
Test–retest (Pearson’s correlation)	NA	(n = 32) 0.78	(n = 22) 0.72

NA = Not applicable

**Table 5:** Individual Item Analysis of Questions with Significant Variation Between Groups.

Item	General public (%)	Patient (%)	Pharmacist (%)
Why has your doctor prescribed you this medicine?	7.9	89.6	100
How does this medicine work in your body?	10.5	70.8	100
How many times a day do you need to take this medicine?	10.5	91.7	100
For how long do you need to take this medicine (for example, 3 months, and 6 months, life-long)?	10.5	91.7	100
Is it appropriate to stop taking this medicine once you feel better?	47.4	100	100
Is there any benefit in taking more of this medicine than your doctor has told you to take?	47.4	93.8	95.0
What is the most important side effect of this medicine?	2.6	60.4	100
What is the best step to take if you accidentally take too much of this medicine?	28.9	75.0	100
VKA (warfarin)-specific questions			
What is your target INR range?	0	93.3	95.0
What was your last INR reading?	0	93.3	95.0
Are regular INR tests necessary to know how well this medicine is working?	21.1	100	90.0
Is an INR value above your target range good for your general wellbeing?	2.6	66.7	90.0
Is it possible for INR values below your target range to be bad for your health?	10.5	80.0	87.5
Is it possible for what you eat to affect your warfarin therapy?	21.1	73.3	92.5

Although this study was not designed to assess the differences in test scores based on educational level, analysis of the general public group indicated that high school education or less was significantly associated with lower performance (p< 0.01). No other differences were observed based on any other demographic characteristics across the three groups.

## Discussion

We have described the development and validation of the Anticoagulation Knowledge Tool (AKT)—an instrument that allows for differences in anticoagulation knowledge to be measured that is applicable to patients taking both the VKAs and DOACs. The AKT is a 20-item knowledge questionnaire with eight additional questions for people taking VKAs (warfarin). Participants in the study were able to complete the survey independently, following written instructions, suggesting that the survey can be self-administered in routine clinical practice like existing tools such as the OAK and AKA. However, unlike the OAK and AKA, our AKT incorporates both open ended and multiple choice questions, as surveys with only multiple choice questions have the disadvantage of providing clues to the correct answers and increasing patients’ total score [15]. Participants who completed the survey on the spot spent between 10–15 minutes, while the length of time for participants who prefer to use the reply paid envelope option could not be ascertained. This suggest that the questionnaire can be completed in a relatively short period of time.

The method used in this study is consistent with recent consensus for the development and validation of new instruments. For content validity, a number of methods have been proposed for the content validation of new instruments including the T index (Tinsley & Weiss, 1975); Content validity ratio ‘CVR’ (Lawshe, 1975); rWG index (James et al, 1984); CVI (Lynn, 1986) and r*WG index (Lindell et al, 1999) [16–20]. The CVI was used in this study as it has the advantages of being easy to compute, easy to understand, focusing on both agreement of relevance among experts and consensus (proportion in agreement) rather than consistency (extent to which experts are consistent in their application of the rating scale), and providing both item and scale level information [11, 19, 21]. The S-CVI value of 0.92 obtained is above the recommended standard of 0.8 for new scales. Furthermore, the majority of items had a modified kappa statistic that corresponded to either the ‘good’ or ‘excellent’ rating; only one item had ‘fair’ rating of 0.5. This suggests that agreement on relevance of each question was not due to chance and, overall, items were highly representative of the underlying construct.

For construct validity, the result of the one-way ANOVA with post-hoc analysis showed a statistically significant difference across the three groups. This result is in agreement with the underlying principle for the group comparison method for construct validity of a new instrument [13], and it therefore follows that the instrument may be useful in distinguishing between different levels of anticoagulation knowledge. The significant variation observed with some items after the individual item analysis further supports the difference in knowledge across the three groups. This may imply that these items would be useful in routine clinical practice as a quick approach in identifying patients with low levels of anticoagulation knowledge. The internal consistency and test-retest reliability coefficients were also acceptable. For the internal consistency reliability analysis, values of > 0.70 obtained across the three groups suggest that the items in the test are interrelated and of a reasonable length, and also measuring the same construct [14]. Further, the result of the test-retest reliability showed correlation coefficients of 0.78 and 0.72 in the patient and pharmacist group, respectively. There has been some debate on the acceptable level for test-retest reliability due to varying statistical techniques, however, a recent systematic review considered a minimum reliability threshold of 0.7 as being adequate [22]. This suggests that the scale is expected to provide consistent scores over time in a stable population.

Participants in the patient group in the validation study scored a mean score of 62% on the AKT. This result is similar to those reported in prior studies. A mean score of 64% was recorded by Winans et al in inpatients new to warfarin therapy [23], while Tang et al reported a mean score of 48% in patients attending an anticoagulation clinic for at least 2 months [7]. Similarly, Davis et al and Hu et al have also reported that less than 40% of patients in routine clinical practice have adequate anticoagulation knowledge [5, 24]. These results suggest that there remains a significant gap in patient anticoagulation knowledge in contemporary practice, and further investigation in a larger cross-section of people taking oral anticoagulants is warranted.

Another important observation in the patient group is that participants taking the DOACs were less likely to view skipping a dose of their medication as a problem compare to participants taking warfarin. This is a critical knowledge gap because the DOACs have shorter half-lives compare to warfarin, and non-adherence to therapy even for a short period can result in loss of clinical effect and expose patients to significant risk [25]. This suggests that significant attention should be given to the concept of medication adherence when designing and implementing an educational intervention in patients prescribed the DOACs.

### Limitations

Among participants in the general public group, about 70% had formal education beyond high school level, including 28% with a post-graduate qualification. The high literacy level of this group may not be truly representative of the general public. However, the average score of this group was still significantly lower than both the patient and pharmacist groups. Also, participants in the three groups were not aged matched, and it is not known if a higher median age in the general public group would have given a higher result. All the participants in the survey were given the opportunity of either completing the survey immediately upon receipt or returning it using a reply paid envelope; we cannot rule out the possibility that some participants might have accessed additional resources despite being encouraged not to do so in the survey instructions. This may have increased the overall score in the survey. The relatively high score in the patient group may be as a result of the recruitment of confident and enthusiastic patients who have had or are undergoing some form of educational training on the use of oral anticoagulant medication, and may not necessary reflect the broader anticoagulant-medication taking population. For the test-retest reliability, not all the participants who completed the first test participated in the second test, and the impact of this on the test-retest reliability coefficient remains unknown. Lastly, the study was conducted in a single region, and the instrument may need to be validated in other regions globally.

## Conclusion

To the best of our knowledge, the AKT is the first validated instrument that can be employed in assessing anticoagulation knowledge of patients taking either the VKAs or the DOACs. It appears to be a valid and reliable instrument in assessing different levels of anticoagulation knowledge. Therefore, it could be useful in routine clinical practice for determining gaps in patients’ anticoagulation knowledge, measuring changes in anticoagulation knowledge over a period of time or in response to educational interventions, and in clinical research for determining the association between anticoagulation knowledge and health related outcomes.

## Supporting Information

S1 Appendix Anticoagulation Knowledge Tool. (DOCX)

## References

[1] Dentali F, Riva N, Crowther M, Turpie AG, Lip GY, Ageno W. Efficacy and safety of the novel oral anticoagulants in atrial fibrillation: a systematic review and meta-analysis of the literature. Circulation. 2012:CIRCULATIONAHA. 112.115410. 10.1161/CIRCULATIONAHA.112.115410 23071159

[2] Jin J, Sklar GE, Min Sen Oh V, Chuen Li S. Factors affecting therapeutic compliance: A review from the patient’s perspective. Therapeutics and clinical risk management. 2008;4(1):269–86. 18728716 10.2147/tcrm.s1458 PMC2503662

[3] Baker JW, Pierce KL, Ryals CA. INR goal attainment and oral anticoagulation knowledge of patients enrolled in an anticoagulation clinic in a Veterans Affairs medical center. Journal of managed care pharmacy: JMCP. 2011;17(2):133–42. 21348546 10.18553/jmcp.2011.17.2.133 PMC10438327

[4] Taylor FC, Ramsay ME, Tan G, Gabbay J, Cohen H. Evaluation of patients' knowledge about anticoagulant treatment. Quality in Health Care. 1994;3(2):79–85. 10137589 10.1136/qshc.3.2.79 PMC1055201

[5] Davis NJ, Billett HH, Cohen HW, Arnsten JH. Impact of adherence, knowledge, and quality of life on anticoagulation control. The Annals of pharmacotherapy. 2005;39(4):632–6. 15713790 10.1345/aph.1E464

[6] Kagansky N, Knobler H, Rimon E, Ozer Z, Levy S. Safety of anticoagulation therapy in well-informed older patients. Archives of internal medicine. 2004;164(18):2044–50. 15477441 10.1001/archinte.164.18.2044

[7] Tang EOY, Lai CS, Lee KK, Wong RS, Cheng G, Chan TY. Relationship between patients’ warfarin knowledge and anticoagulation control. Annals of Pharmacotherapy. 2003;37(1):34–9. 12503930 10.1345/aph.1A198

[8] Yahaya A, Hassali M, Awaisu A, Shafie A. Factors associated with warfarin therapy knowledge and anticoagulation control among patients attending a warfarin clinic in Malaysia. J Clin Diag Res. 2009;3:1663–70.

[9] Briggs AL, Jackson TR, Bruce S, Shapiro NL. The development and performance validation of a tool to assess patient anticoagulation knowledge. Research in social and administrative Pharmacy. 2005;1(1):40–59. 17138465 10.1016/j.sapharm.2004.12.002

[10] Zeolla MM, Brodeur MR, Dominelli A, Haines ST, Allie ND. Development and validation of an instrument to determine patient knowledge: the oral anticoagulation knowledge test. Annals of Pharmacotherapy. 2006;40(4):633–8. 16551766 10.1345/aph.1G562

[11] Polit DF, Beck CT, Owen SV. Is the CVI an acceptable indicator of content validity? Appraisal and recommendations. Research in nursing & health. 2007;30(4):459–67. 17654487 10.1002/nur.20199

[12] Polit DF, Beck CT. The content validity index: are you sure you know what's being reported? Critique and recommendations. Research in nursing & health. 2006;29(5):489–97. 16977646 10.1002/nur.20147

[13] Westen D, Rosenthal R. Quantifying construct validity: Two simple measures. Journal of Personality and Social Psychology. 2003;84(3):608 12635920 10.1037//0022-3514.84.3.608

[14] Tavakol M, Dennick R. Making sense of Cronbach's alpha. International Journal of Medical Education. 2011;2:53–5. 28029643 10.5116/ijme.4dfb.8dfd PMC4205511

[15] Thayn KS. An evaluation of multiple choice test questions deliberately designed to include multiple correct answers. 2010.

[16] Tinsley HE, Weiss DJ. Interrater reliability and agreement of subjective judgments. Journal of Counseling Psychology. 1975;22(4):358.

[17] Lawshe CH. A quantitative approach to content validity1. Personnel psychology. 1975;28(4):563–75.

[18] James LR, Demaree RG, Wolf G. Estimating within-group interrater reliability with and without response bias. Journal of applied psychology. 1984;69(1):85.

[19] Lynn MR. Determination and quantification of content validity. Nursing research. 1986;35(6):382–6. 3640358

[20] Lindell MK, Brandt CJ, Whitney DJ. A revised index of interrater agreement for multi-item ratings of a single target. Applied Psychological Measurement. 1999;23(2):127–35.

[21] Hawkins RJ, Swanson B, Kremer MJ, Fogg L. Content Validity Testing of Questions for a Patient Satisfaction With General Anesthesia Care Instrument. Journal of Perianesthesia Nursing. 2014;29(1):28–35. 10.1016/j.jopan.2013.05.011 24461280

[22] Paiva CE, Barroso EM, Carneseca EC, de Pádua Souza C, dos Santos FT, López RVM, et al A critical analysis of test-retest reliability in instrument validation studies of cancer patients under palliative care: a systematic review. BMC medical research methodology. 2014;14(1):1. 24447633 10.1186/1471-2288-14-8 PMC3899385

[23] Winans ARM, Rudd KM, Triller D. Assessing anticoagulation knowledge in patients new to warfarin therapy. Annals of Pharmacotherapy. 2010;44(7–8):1152–7. 10.1345/aph.1P092 20571105

[24] Hu A, Chow C-M, Dao D, Errett L, Keith M. Factors influencing patient knowledge of warfarin therapy after mechanical heart valve replacement. Journal of Cardiovascular Nursing. 2006;21(3):169–75. 16699355 10.1097/00005082-200605000-00003

[25] Mekaj YH, Mekaj AY, Duci SB, Miftari EI. New oral anticoagulants: their advantages and disadvantages compared with vitamin K antagonists in the prevention and treatment of patients with thromboembolic events. Therapeutics and clinical risk management. 2015;11:967 10.2147/TCRM.S84210 26150723 PMC4485791

